# Leptin Promotes the Proliferation and Neuronal Differentiation of Neural Stem Cells through the Cooperative Action of MAPK/ERK1/2, JAK2/STAT3 and PI3K/AKT Signaling Pathways

**DOI:** 10.3390/ijms242015151

**Published:** 2023-10-13

**Authors:** Ruolan Tan, Xiaoxuan Hu, Xinyi Wang, Meiqi Sun, Zhenlu Cai, Zixuan Zhang, Yali Fu, Xinlin Chen, Jing An, Haixia Lu

**Affiliations:** 1 Department of Neurobiology, School of Basic Medical Sciences, Xi’an Jiaotong University Health Science Center, Xi’an 710061, China; tanruolan19930117@163.com (R.T.); xiaoxuanhu21@163.com (X.H.); wangxy_95095@163.com (X.W.); smqmina44@163.com (M.S.); 15339051098@163.com (Z.C.); zhangzixuan_dsa@163.com (Z.Z.); f19965482830@163.com (Y.F.); chenxl@mail.xjtu.edu.cn (X.C.); 2 Department of Human Anatomy and Histo-Embryology, School of Basic Medical Sciences, Xi’an Jiaotong University Health Science Center, Xi’an 710061, China

**Keywords:** neural stem cells, leptin, proliferation, neuronal differentiation, JAK2/STAT3 pathway, PI3K pathway, MAPK pathway

## Abstract

The potential of neural stem cells (NSCs) for neurological disorders the treatment has relied in large part upon identifying the NSCs fate decision. The hormone leptin has been reported to be a crucial regulator of brain development, able to influence the glial and neural development, yet, the underlying mechanism of leptin acting on NSCs’ biological characteristics is still poorly understood. This study aims to investigate the role of leptin in the biological properties of NSCs. In this study, we investigate the possibility that leptin may regulate the NSCs’ fate decision, which may promote the proliferation and neuronal differentiation of NSCs and thus act positively in neurological disorders. NSCs from the embryonic cerebral cortex were used in this study. We used CCK-8 assay, ki67 immunostaining, and FACS analysis to confirm that 25–100 ng/mL leptin promotes the proliferation of NSCs in a concentration-dependent pattern. This change was accompanied by the upregulation of p-AKT and p-ERK1/2, which are the classical downstream signaling pathways of leptin receptors b (LepRb). Inhibition of PI3K/AKT or MAPK/ERK signaling pathways both abolished the effect of leptin-induced proliferation. Moreover, leptin also enhanced the directed neuronal differentiation of NSCs. A blockade of the PI3K/AKT pathway reversed leptin-stimulated neurogenesis, while a blockade of JAK2/STAT3 had no effect on it. Taken together, our results support a role for leptin in regulating the fate of NSCs differentiation and promoting NSCs proliferation, which could be a promising approach for brain repair via regulating the biological characteristics of NSCs.

## 1. Introduction

Leptin is a multifunctional hormone mainly secreted by the white adipocyte tissue and has an effect on regulating appetite and energy homeostasis by activating specific nuclei of the hypothalamus [1]. Leptin exerts physiological effects mainly by binding to its subunit receptors b (LepRb). When the components bind, it increases Janus-2 kinase phosphorylation and promotes activation of various intracellular signaling molecules, including signal transducer and activator of transcription 3 (STAT3), MAPK/ERK1/2 and the PI3K/AKT pathway proteins.

In addition to its well-established regulation on energy homeostasis and food intake, a developmental role of leptin is suggested by the production of leptin by the brain itself. Leptin mRNA and proteins could be detected in brain regions with high expression of LepRs in both rats and mice [2]. Furthermore, LepRs were identified in the mouse embryo as early as embryonic day (E) 10.5 [3]. In *ob/ob* mice, deficiency of leptin leads to reduced brain weight, structural abnormalities of neurons, decreased total brain protein content, and impaired myelination [4,5,6,7,8]. However, leptin administration could normalize the brain weight and total protein content, as well as the locomotor activity [9]. These studies reveal that leptin may play an important role in brain development and neurogenesis. Leptin has been shown to be neuroprotective in various neurological disorders [10,11,12,13,14]. Amantea et al. found that acute administration of leptin could significantly reduce the brain infract and neurological deficit in a permanent ischemia model of rodents, and that STAT3 phosphorylation in astrocyte and neurons is responsible for its positive function [15]. Furthermore, similar experiments have demonstrated that PI3K/AKT is beneficial for the survival of neurons in the ischemic penumbra, and could rescue these cells from death [16]. However, the underlying mechanism of leptin in the fate decision of NSCs is still poorly understood.

NSCs hold promise for the treatment of neural injury and neurodegenerative diseases depending on its renewal and neuronal differentiation [17,18,19]. However, the availability of the cells, and their amplification and manipulation in vitro prior to intra-CNS transplantation, limits their usage [20]. Therefore, experimental strategies designed to keep NSCs’ viability and differentiation fate may lead to novel therapeutic approaches to neurodegenerative or demyelination diseases. A large number of studies have been conducted on the fate determination of NSCs, among which the mechanisms of multiple nutritional factors and microenvironment on the proliferation and fate determination of NSCs deserve attention [21]. Some exciting data showed that leptin levels have a two to three fold increase during pregnancy in humans, and surge between the first weeks in postnatal-development of rodents, which coincides with neuronal growth and synaptogenesis [22,23,24,25,26,27]. Thus, there is growing evidence that leptin may play a critical role in the biological characteristics of NSCs.

In the current study, we cultured NSCs derived from SD rat embryos in vitro and observed their biological behaviors after leptin treatment. We found that leptin has a neurogenic potential effect on NSCs, and may regulate the proliferation and neuronal differentiation of NSCs via MAPK/ERK and PI3K/AKT pathways. It would provide evidence for leptin as a potential supportive therapeutic efficacy for an NSCs treatment strategy.

## 2. Results

### 2.1. LepRs Were Wildly Expressed in NSCs

**Figure 1:**
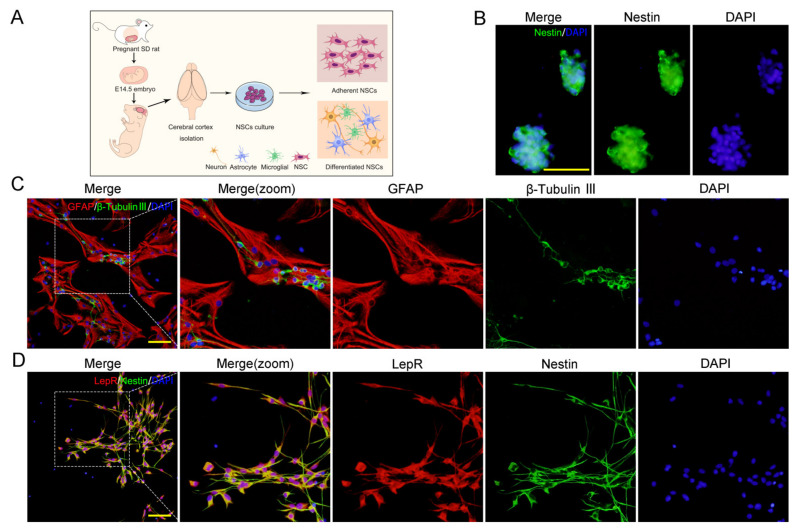
Identification and LepRs expression of NSCs. (**A**) Experimental schematic for collection and cultivation of NSCs. (**B**,**C**) Immunofluorescence results for marker proteins (Nestin, β-tubulin III, and GFAP) that were used to identify the self-renewal and multipotential of NSCs. Scale bar represents as 100 μm (**B**) and 50 μm (**C**,**D**) NSCs were assessed using double-labelled immunofluorescence for Nestin (green) and LepRs (red). Scale bar = 50 μm.

NSCs were isolated from E14.5 cerebral cortex of SD rat embryos and cultured in complete medium (CM) (Figure 1A). Extensive immunostaining for NSC markers (Nestin) was observed in neurospheres after 4–5 days culture (Figure 1B). After being maintained in differentiation medium (DM) for 7 days, those cells mainly differentiated into β-tubulin III^+^ neurons and GFAP^+^ astrocytes (Figure 1C). These results confirmed that the cells we harvested are NSCs.

Previous studies have indicated that the intracellular effects of leptin are mediated by the leptin receptor [28]. It was observed that more than 90% of the Nestin-positive NSCs expressed LepRs (Figure 1D).

### 2.2. Leptin Promoted Cell Proliferation and Increased Cell Viability

**Figure 2:**
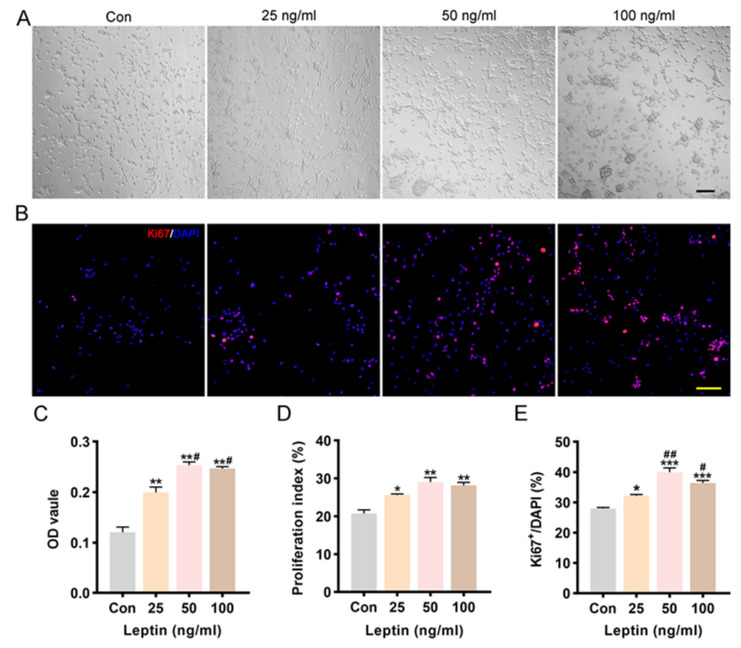
Leptin promotes NSCs proliferation and increases cell viability. (**A**) Representative images of light microscope of NSCs cultured in 96-well plate after 48 h leptin treatment. Compared with control group, leptin significantly promoted cell aggregation and number. Scale bar = 100 μm. (**B**,**E**) Ki67 staining showed proliferating NSCs (red) cultured in control and leptin-contained growth media growing for 3 days. Scale bar = 100 μm, (**B**). Bar graph showed the percentage of Ki67 positive cells (red) to DAPI staining cells (blue) in each group (**E**). Quantification of ki67+ cells as a percentage of total cells in leptin group was significantly increased compared to control. (**C**) OD450 value of NSCs in growth media was measured at third day after leptin application. Viability of NSCs significantly increased after leptin application. (**D**) FACS evaluated cell cycle of NSCs cultured in control and different concentration of leptin. The percentage of PI in leptin was dramatically increased vs. control group. ANOVA test * *p* ˂ 0.05, ** *p* ˂ 0.01, *** *p* ˂ 0.001 vs. control group, # *p* ˂ 0.05, ## *p* ˂ 0.01 vs. Leptin (25 ng/mL) group.

It has been reported that leptin maintained neurosphere cells via increasing the ratio of viable colony number to plated cell number in a dose-dependent manner [29]. In the current study, P2 NSCs were incubated with different concentrations of leptin (25, 50, 100 ng/mL), and then cell viability and proliferation were observed at 3DIV. In this study, cell viability was dramatically promoted after leptin treatment for 48 h, as shown by the OD value of the CCK-8 Assay (Figure 2C). Compared with controls, there was a concentration-dependent effect of leptin on cell viability at low dose. However, the effect of 100 ng/mL leptin was similar to that of 50 ng/mL leptin. The cell viability of the 50 ng/mL leptin treatment group was slightly higher than that of the higher concentration (100 ng/mL) treatment group.

Furthermore, proliferation was investigated by calculating the proliferation index (PI) and percentage of ki67-positive cells. After being treated with leptin for 48 h, the proliferation index showed a significant increase in a concentration-dependent manner of NSCs’ proliferation (Figure 2D). Meanwhile, the percentage of Ki67^+^ cells in the leptin treatment group also increased significantly compared to the control group (Figure 2B,E). There was no significant difference between 50 and 100 ng/mL leptin in promoting proliferation at 3DIV, we choose the optimal concentration of 50 ng/mL leptin for use in the following experiments. Observation of the light microscope images showed that cell densities (Figure 2A) increased in a concentration-dependent manner and showed a tendency to aggregate. Together, it showed that leptin contributes to NSCs proliferation with a concentration-dependent effect.

### 2.3. MAPK/ERK 1/2 and PI3K/AKT Involves in Leptin-Induced Survival and Proliferation of NSCs

**Figure 3:**
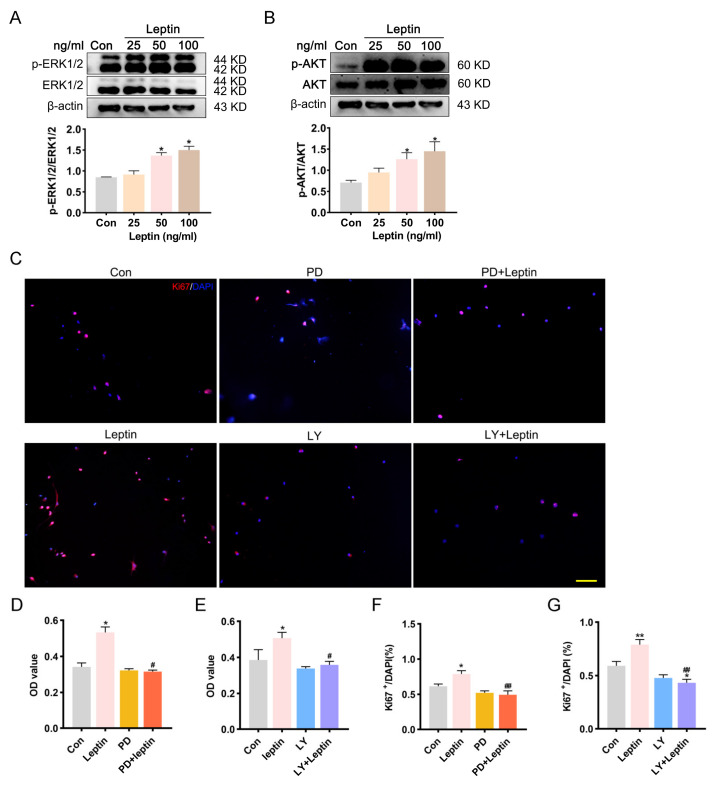
PI3K/Akt and MAPK/ERK signaling pathways are involved in leptin-induced proliferation of NSCs. (**A**,**B**) Western blot analysis of the level of the p-ERK and p-AKT protein derived from proliferating NSCs in control and leptin (50 ng/mL) groups on the third day. The ratio of p-ERK/ERK (**A**) and p-AKT/AKT (**B**) was increased significantly in response to leptin treatment compared with the control group. (**C**,**F**,**G**) ki67 immunostaining showed that, compared with the leptin group, the percentage of Ki67 positive cells (red) was significantly reduced after PD98059 and LY294002 pretreatment. (**D**,**E**) Administration of PD98059 and LY294002 significantly reversed the effect of leptin on cell viability. PD: PD98059, inhibitor for MAPK; LY: LY294002, inhibitor for PI3K. Scale bar = 100 μm. All comparisons were made using ordinary one-way ANOVA and LSD multiple comparisons. Significance: * p ˂ 0.05, ** *p* ˂ 0.01 vs. control group, # *p* ˂ 0.05, ## *p* ˂ 0.01 vs. Leptin (50 ng/mL) group. LY: LY294002, PD: PD98059.

PI3K/AKT and MAPK/ERK signaling pathways play key roles in controlling cell proliferation, survival, and motility [30]. LepRb can activate downstream tyrosine kinase cascades, including the PI3K/AKT and ERK pathways [31,32,33,34]. Therefore, whether leptin can promote NSCs viability and proliferation via PI3K/AKT and ERK pathways had been detected. The activation of PI3K/AKT and ERK signaling pathways was affected by increasing phosphorylation levels of ERK 1/2 and AKT after 48 h leptin administration (Figure 3A,B).

To further confirm the role of ERK and PI3K/AKT signaling pathways in leptin promoting NSCs proliferation effect, corresponding inhibitors (PD98059 for ERK, LY294002 for AKT) were used. Cells were pre-incubated with PD98059 or LY294002 for 2 h before leptin treatment. Treatment with PD98059 or LY294002 counteracted the leptin effect on proliferation, as indicated by ki67 staining (Figure 3C,D) and OD values measured by the CCK8 assay (Figure 3E). Taken together, the promoting survival and proliferation of NSCs induced by leptin were significantly abolished by specific blockades of both MAPK/ERK1/2 and PI3K/AKT signaling.

### 2.4. PI3K/AKT and JAK2/STAT3 Signaling Pathways Contribute to Leptin-Induced Neuronal Differentiation In Vitro

**Figure 4:**
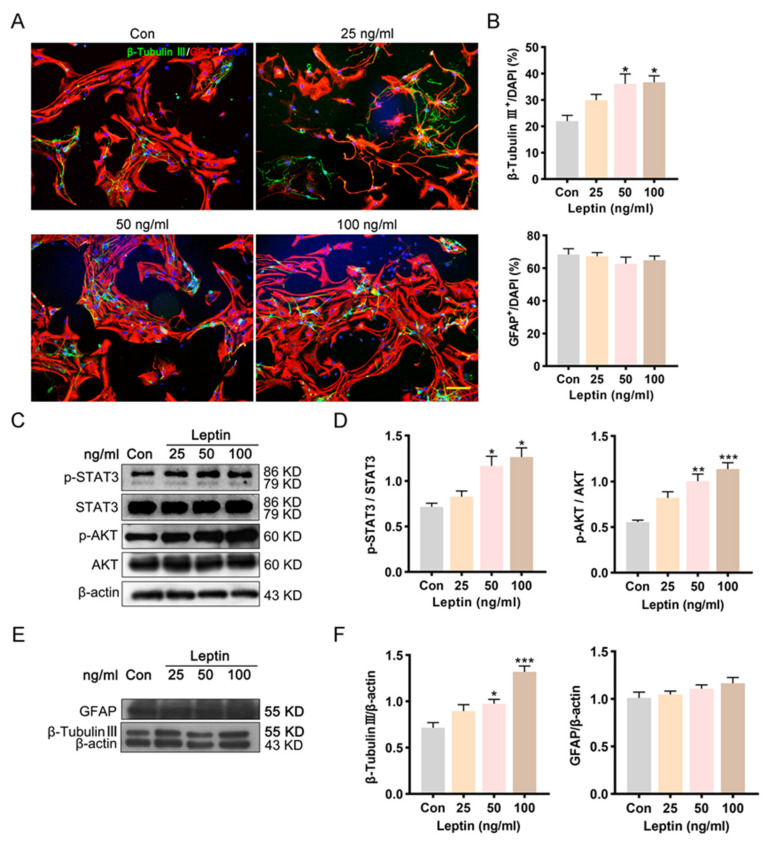
Leptin promotes NSCs neuronal differentiation and activates PI3K/AKT and JAK2/STAT3 pathways. (**A**,**B**) Proportion of β-tubulin III^+^ neurons (green) and GFAP^+^ astroglial (red) after 7 days of in vitro differentiation. Compared with the control, the proportion of β-tubulin III^+^ cells was significantly increased in 50 and 100 ng/mL leptin groups. (**C**,**D**) Western blot analysis of the level of p-STAT3 and p-AKT protein derived from differentiated NSCs after 24 h administration of leptin. (**E**,**F**) Western blot analysis showed that β tubulin III protein levels were significantly elevated after leptin stimulation, while GFAP protein levels had no significant difference. Scale bar = 100 μm. All comparisons were made using ordinary one-way ANOVA and LSD multiple comparisons. Significance: * *p* ˂ 0.05, ** *p* ˂ 0.01, *** *p* ˂ 0.001 vs. control group.

Leptin regulates neurogenesis and neurocircuits formation before and after birth, which are required for normal development of forebrain pathways [35,36]. The STAT3 signaling pathway is found to be important for the maintenance of neural development [37,38]. However, it was unclear whether PI3K/AKT and JAK2/STAT3 play roles in leptin regulated NSCs’ differentiation. In this study, NSCs were treated with leptin and cultured for 7 days in DM. Then, the percentages of β-tubulin III and GFAP positive cells were calculated (Figure 4A). The proportions of β-tubulin III^+^ cells in the 50 and 100 ng/mL leptin group were significantly increased compared with the control group (* p < 0.05), while the low concentration (25 ng/mL) had no difference (Figure 4B). No significant difference in the percentage of GFAP^+^ was found between each group (Figure 4B). To determine the activation of PI3K/AKT and JAK2/STAT3 signaling pathways, phosphorylation levels of AKT on Ser473, and STAT3 on Try705 were evaluated by Western blot following leptin treatment at different doses. After leptin treatment for 24 h, the level of p-STAT3 showed a dose-dependent elevation (* *p* < 0.05; Figure 4C,D). AKT showed a low level of basal phosphorylation in cultured NSCs, and leptin treatment for 24 h increased AKT phosphorylation level (* *p* < 0.05; Figure 4C,D). These results suggested that the PI3K/AKT and JAK2/STAT3 signaling pathways are recruited by leptin and may be involved in NSCs differentiation.

**Figure 5:**
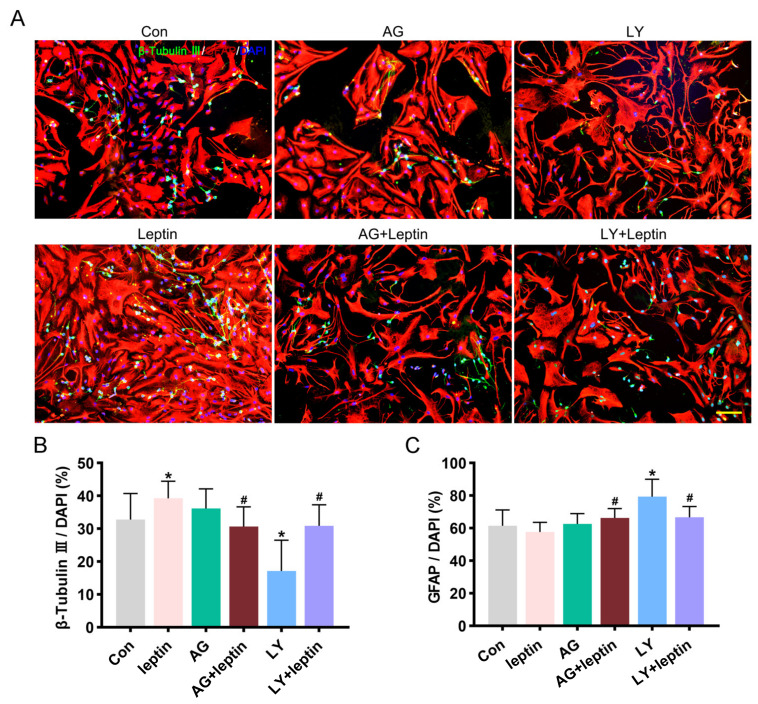
Leptin differentially regulates the proportion of neurons and astrocytes through PI3K/AKT and JAK2/STAT3 signaling pathways. (**A**) Representative images of NSCs differentiation, cells were double-stained with β-tubulin III and GFAP (neuron in green and astrocyte in red; scale bar = 100 μm). NSCs were incubated with LY294002 (20 μM) or AG490 (10 μM) for 2 h followed by treatment with leptin (50 ng/mL) for 7 days. (**B**) Quantitative analysis revealed that LY + Leptin or AG + Leptin groups both abolished the increase in percentage of neurons vs. control. However, the LY group significantly reduced the percentage of neurons, while the AG group had no effect vs. control. (**C**) Quantitative analysis revealed that LY + Leptin or AG + Leptin group both elevated the ratio of astrocytes. The LY group also significantly increased the ratio of astrocytes. All comparisons were made using ordinary one-way ANOVA and LSD multiple comparisons. Significance: * p ˂ 0.05 vs. control group, *# p* ˂ 0.05 vs. Leptin (50 ng/mL) group. AG: AG490, LY: LY294002.

The NSCs were exposed to AKT inhibitor LY294002 (20 µM) or STAT3 inhibitor AG490 (10 μM), for 2 h before leptin treatment. Double immunofluorescence staining of GFAP and β-tubulin III showed that pretreatment with AG490 or LY294002 both abolished the elevated percentage of β-tubulin III by leptin (Figure 5A). Compared with the leptin group, the proportion of neurons decreased significantly in the AG + Leptin group or the LY + Leptin group (*# p* < 0.05; Figure 5B), while the proportion of astrocytes was significantly up-regulated (*# p* < 0.05; Figure 5C). The AG490 group did not change the proportion of astrocytes; the proportion of neurons was modestly increased but not statistically significant. Interestingly, the LY294002 group greatly inhibited the proportion of neurons and promoted the proportion of astrocytes compared with the control group (* p < 0.05; Figure 5B,C).

**Figure 6:**
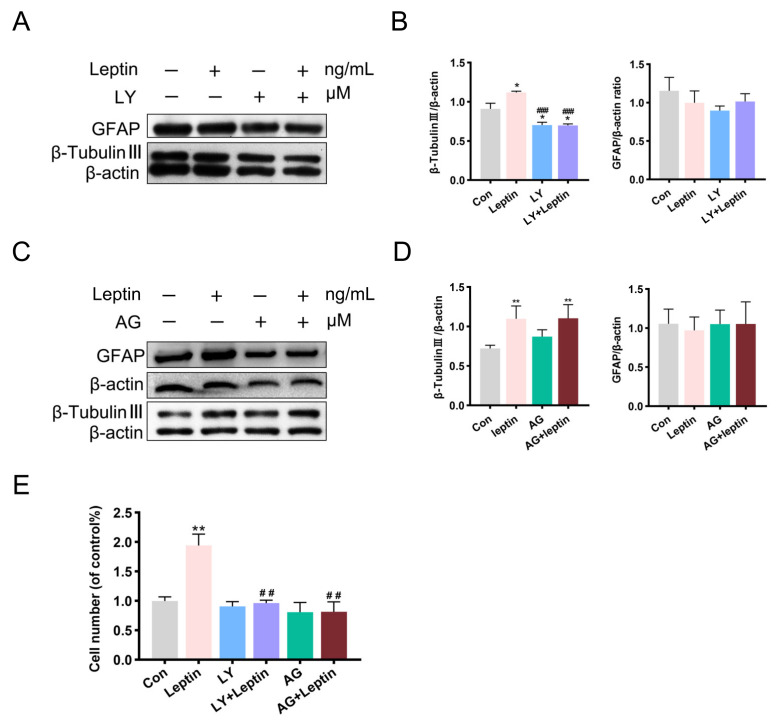
Leptin promotes the directed differentiation of NSCs into neurons through the PI3K/AKT signaling pathway. (**A**,**B**) The expression of β-tubulin III was significantly reduced with LY294002 pre-treatment; There were no significant differences in the GFAP protein levels between groups. (**C**,**D**) Western blot analysis showed that the β-tubulin III protein level was significantly elevated after leptin stimulation. Blocking the JAK2/STAT3 pathway showed no significant effect on leptin-induced β-tubulin III expression; There were no significant differences in the GFAP protein levels between groups. (**E**) Differentiated cells derived from NSCs were counted and statistically analyzed. Cell count analysis showed that leptin significantly increased cell numbers vs. control, when applying with AG490 or LY294002, the number of cells were significantly reduced. All comparisons were made using ordinary one-way ANOVA and LSD multiple comparisons. Significance: * *p* ˂ 0.05, ** *p* ˂ 0.01 vs. control group, ## *p* ˂ 0.05, ### *p* ˂ 0.001 vs. Leptin (50 ng/mL) group. AG: AG490, LY: LY294002.

Consistent with the staining results, the protein level of β-tubulin III was significantly increased after leptin treatment, while pretreatment with LY294002 reversed this effect (### *p* < 0.001, Figure 6A,B). In addition, compared with the control, LY294002 treatment alone also significantly reduced the protein level of β-tubulin III. Interestingly, inhibition of the JAK2/STAT3 pathway showed no significant effect on leptin-induced β-tubulin III expression (** *p* < 0.01, Figure 6C,D). 

Unlike its result on astrocyte proportion in staining, the level of GFAP protein seems to follow a different pattern. No significant difference was found in the level of GFAP protein between groups (Figure 6B,D).

Total cell numbers of the differentiated cells were also counted in each group, the results revealed that leptin caused a roughly two-fold increase in the total cell counts, and inhibition of PI3K or JAK2 both reversed the effect (Figure 6E).

It was presented that leptin increased the number of differentiated cells and promoted neuronal differentiation. The PI3K/AKT pathway seemed directly involved in neuronal differentiation of NSCs.

## 3. Discussion

Leptin plays a key role in brain development and acts as a neuroprotector for brain injury [39,40]. NSCs were considered as a promising strategy for the treatment of brain injury and neurodegenerative diseases [41]. However, the effect of leptin on exogenous NSCs is unclear. In this study, the alteration of biological characters of exogenous NSCs was explored after leptin treatment. The aim was to study the potential application of leptin on NSCs therapy.

It was reported that long-term administration of leptin could rescue the age and genotype-dependent reduced proliferation of adult SVZ and SGZ cells and increased the expression of Nestin [42]. We demonstrated that leptin directly stimulated proliferation of exogenous NSCs. The mitogenic effect of leptin was supported by CCK-8 assay as well as immunostaining for the proliferative marker Ki67. In addition, cell cycle analysis of NSCs showed a significant increase in the proliferation index. The results were consistent with the maintenance of embryonic cortical neurosphere proliferation by leptin [29,43]. Although leptin functions as growth factors in various cell types, the specific mechanism of leptin-induced proliferation of NSCs is not fully understood. Leptin induces proliferation and (or) anti-apoptosis effect of breast cancer cells and vascular smooth muscle cells by activating the PI3K pathway [44,45]. The ERK1/2 pathway has also been shown to play an important role in regulating the proliferation of a variety of stem cells [46,47,48]. In this study, it was found that leptin-induced NSCs proliferation was accompanied by an increase in phosphorylation of AKT and ERK1/2. When treated with the specific signaling inhibitors of AKT or ERK1/2, this leptin-induced proliferation was abolished. These findings suggested that leptin may induces NSCs proliferation via MAPK/ERK1/2 and PI3K/AKT pathways.

Next, we investigated the effect of leptin on neural stem cell differentiation. Leptin could enhance the proportion of neurons. And the phosphorylation levels of STAT3 and AKT were increased, indicating that these two pathways were further activated for neuronal differentiation, while the proportion of astrocytes did not have any difference. Western blot analysis showed that leptin treatment significantly increased the expression of neuronal marker β-tubulin III, but the expression of astrocyte marker GFAP did not significantly change.

The JAK/STAT signaling pathway participates in survival, proliferation, apoptosis, and differentiation of NSCs. The effect of STAT3 on NSCs function is multifaceted [49,50,51]. STAT3 has been proved to have an important relationship with the survival and regeneration of neurons in various central nervous system injury models such as cerebral ischemia injury and spinal cord injury [50]. To further explore the role of JAK2/STAT3 in NSCs differentiation, cells were pre-treated with AG490, a JAK2 specific inhibitor. Immunostaining results showed that AG490 pre-treatment reversed the effect of leptin on NSCs differentiation and reduced the proportion of neurons compared with the leptin group. AG490 treatment alone had no effect on the proportion of neurons. Interestingly, the results of Western blot did not support this; the protein level of β-tubulin III in the AG490 and leptin combination group had no difference compared with the leptin group. Also, GFAP protein levels did not differ between groups. It seems that JAK2/STAT3 was not responsible for the direct regulation in leptin-induced neuronal differentiation.

Additionally, our results showed that PI3K/AKT was directly involved in leptin regulation of NSCs differentiation. When leptin was co-administrated with LY294002, the proportion of neurons had a significant decrease compared with the leptin group. Western blot results coincided with the staining results; inhibition of the PI3K/AKT pathway significantly reversed leptin-induced β-tubulin III expression. Moreover, we observed that LY294002 treatment alone also significantly down-regulated the expression of β-tubulin III. However, there is no difference in GFAP expression between each group. These results suggest that the PI3K/AKT pathway is an intrinsic regulatory pathway in the differentiation of neural stem cells into neurons, and it was involved in the leptin-regulated differentiation fate of NSCs.

One particularly interesting finding was the increased number of differentiated cells derived from NSCs. A previous study in mice suggested that leptin–stimulated hippocampal neurogenesis is based on the cell proliferation rather than modifying cells fate [52]. In our present study, JAK2/STAT3 is somehow related to leptin-enhanced NSCs differentiation, in which the increase of leptin on total cell numbers of differentiated is blocked by AG490 or LY294002. These results are partially consistent with our cell counting results.

**Figure 7:**
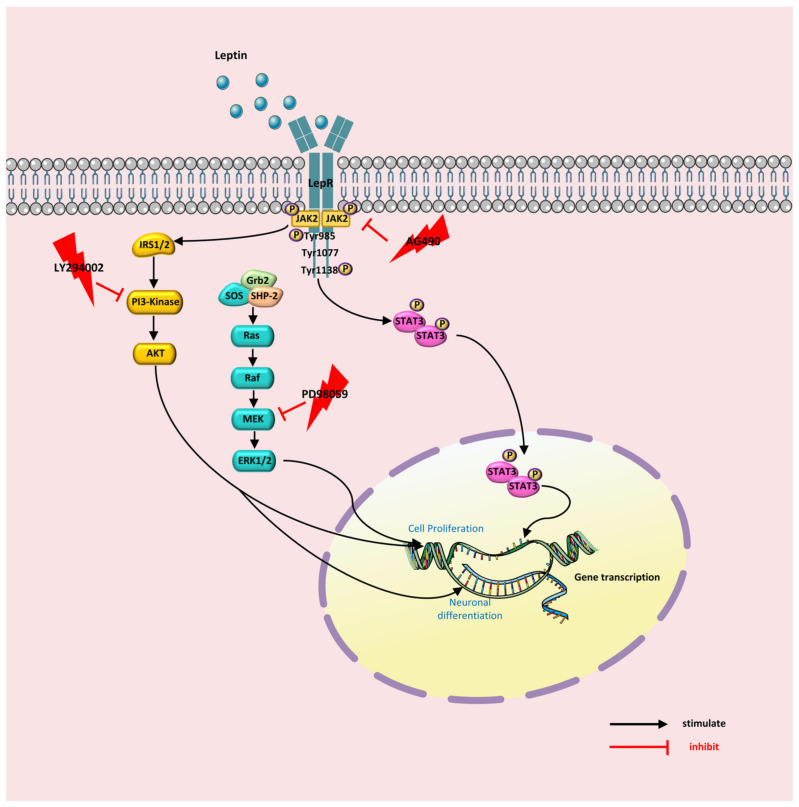
Possible mechanism for leptin regulating the proliferation and neuronal fate of NSCs. Grb2: growth factor receptor-bound protein 2; SOS: Ras activating protein; PI3K: Phosphoinositide 3-kinase; JAK2: Janus kinase; LepR: leptin receptor b; NSC: neural stem cells.

In conclusion, leptin promotes proliferation and neurogenesis of NSCs in vitro. Leptin recruits MAPK/ERK1/2, and PI3K/AKT signaling pathways for NSCs proliferation. The effect of leptin on neuronal differentiation is directly by regulating PI3K/AKT pathways (Figure 7).

## 4. Materials and Methods

### 4.1. Animals

Pregnant Sprague-Dawley (SD) rats (8–10 weeks old) used for NSCs cultures were provided by the Experimental Animal Center, Xi’an Jiaotong University Health Science Center. Rats were maintained under a standard 12 h dark–light cycle in a controlled temperature (22 ± 1 °C) with free access to food and drink. All procedures involving animal work were confirmed and monitored by the Institutional Animal Care and Use Committee (IACUC) of Xi’an Jiaotong University under protocol number 2021-280.

### 4.2. Culture and Identification of Embryonic NSCs

Primary NSCs cultures were prepared from the cerebral cortex of E14.5 embryos. Embryos were obtained from 14.5-day (E14.5) pregnant SD rat, and the cerebral cortices were separated from embryonic brains in the pre-cooled phosphate buffer solution (PBS). After carefully separating the meninges and choroid plexus from the cortex tissue, the tissues were then transferred to the pre-cooled DMEM/F-12 medium (#12400, Gibco Life Technologies, Carlsbad, CA, USA), and quickly minced by ophthalmic micro-tweezers. After gently trituration with a pipette, no chunks of tissue were left. To obtain a single cell suspension, the cell suspensions were filtered using a 40 μm cell strainer (BD Falcon, Franklin Lakes, NJ, USA). Cells were cultured in complete medium (CM) with 10 ng/mL bFGF (#PHG0021, Gibco Life Technologies, Carlsbad, CA, USA), 20 ng/mL EGF (#PHG0311, Gibco Life Technologies, Carlsbad, CA, USA), 200 U/mL penicillin, 100 μg/mL streptomycin, 1% N2 (#17502-048, Gibco Life Technologies, Carlsbad, CA, USA), and 2% B27 (#17504-044, Gibco Life Technologies, Carlsbad, CA, USA) supplement in DMEM/F12 medium at 37 °C under 5% CO_2_ following the standard protocol [53] and optimized in our laboratory [54,55]. After incubation for 5~6 days, cells were passaged. The passage (P) 2 NSCs were mainly used for the following experiments.

P2 NSCs were identified by immunocytochemistry staining. Cells were fixed with 4% paraformaldehyde (PFA) in a 0.1 M sodium phosphate buffer solution (PBS, pH 7.4) for 15 min, then permeabilized with 0.3% TritonX-100 for 10 min and blocked with 5% bovine serum albumin (BSA) for 1 h at room temperature. After blocking, cells were incubated with primary antibodies including anti-Nestin (#NBP1-92717, Novus, Littleton, CO, USA, 1:400), anti-GFAP (1:1000, #NB-300-141, Novus, Littleton, CO, USA), anti-β-tubulin III (#ab78078, abcam, Cambridge, UK, 1:1000) and anti-Lep-Rb (#ab5593, abcam, Cambridge, UK, 1:400) at 4 °C overnight. Then, cells were thoroughly washed with PBS, and incubated with secondary antibodies Alexa Fluor 488 and 594 conjugated donkey anti-rabbit/donkey anti-mouse IgG (#A-21202, Invitrogen, Carlsbad, CA, USA, 1:1000; #A-21207, Invitrogen, Carlsbad, CA, USA, 1:1500) for 2 h. The cell nuclei were stained with DAPI (#H-1200, Vector Laboratories, 0.1 μg/mL) for 10 min at room temperature. Coverslips were mounted and imaged with a fluorescence microscope Olympus BX-57 (Olympus Corporation, Tokyo, Japan).

### 4.3. Viability of NSCs

Cell counting kit-8 (CCK-8, Dojindo, Kumamoto, Japan) was used to investigate cell viability according to the manufacturer’ protocol. After digestion with cell dissociation solution TryPLE Express 1× (Invitrogen, Carlsbad, CA, USA), neurospheres were isolated into single cells, then resuspended with CM and cultured in PLL coated 96-well plates (2 × 10^4^ cells/well). After 24 h, the NSCs were pre-treated with the inhibitors for 2 h, and leptin (#400-21, Peprotech, Rocky Hill, NJ, USA) was then added to the medium to reach a final concentration. The working concentration of leptin were 25, 50, and 100 ng/mL, and/or the inhibitors were applied including the mitogen-activated protein kinase kinase-1 (MEK1) inhibitor PD98059 (#S1177, Selleck, Houston, TX, USA, 20 μM), phosphoinositide 3 kinase (PI3K) inhibitor LY294002 (#S1105, Selleck, Houston, TX, USA, 20 μM)

After another 48 h, about 10 μL cck-8 solution (water-soluble tetrazolium salt WST-8 [2-(2-methoxy-4-nitrophenyl)-3-(4-nitrophenyl)-5-(2, 4-disulfophenyl)-2H tetrazolium]) was added into each well and then incubated at 37 °C. The optical density (OD) of formazan dye was detected by a microplate spectrophotometer (BioTek, Winooski, VT, USA) at 450 nm for 3 h of incubation. Results were averaged for 3 independent experiments, with each experiment containing at least 5 readings.

### 4.4. Proliferation of NSCs

NSCs proliferations were determined by both ki67 immunostaining and flow cytometry. Cells were treated as above with leptin. For cell cycle distribution analysis, cells were collected and washed with cold PBS at 3DIV, then resuspended with 70% ethanol and stored at 4 °C overnight, followed by washing with cold PBS twice. Cells were then resuspended with PBS which contained 100 μg/mL RNase A (R4875, Sigma-Aldrich, St. Louis, MO, USA), 50 μg/mL propidium iodide (P4170, Sigma-Aldrich, St. Louis, MO, USA) and 0.3% Triton X-100 and incubated in the dark at room temperature for 30 min. Samples were detected by flow cytometer (Canto TM, BD Bioscience, Franklin Lakes, NJ, USA). The content of DNA in cells was analyzed with ModFit 161 L T version 3.0 software (Verity Software House, Topsham, Devon, UK) and proliferation index (PI) was calculated by analyzing the percentage of cells in G1, S and G2/M phases. PI = (S + G2/M)/(G0/G1 + S+G2/M) × 100%.

For Ki67 immunostaining, NSCs were seeded on PLL-coated coverslips in 24-well plates (5 × 10^4^ cells/well). The cells were pre-treated with or without the inhibitors (PD98059 & LY294002, 20 μM) for 2 h, and leptin was then added to the medium to reach different final concentrations. At day3, Ki67 (#9129, Millipore, Billerica, MA, USA, 1:400) staining was performed, and the percentage of Ki67^+^ cells in each group was then calculated. The immunocytochemistry staining was performed as above.

### 4.5. Differentiation of NSCs

P2 NSCs were cultured in 24-well plate (5 × 10^4^ cells/well) with differentiation medium (DM) for 24 h and then pre-treated with or without the inhibitors (AG490 (#S114302, Selleck, Houston, TX, USA) & LY294002, 20 μM) for 2 h, and leptin was then added to the medium to reach different final concentrations. The differentiation medium contains DMEM/F12 (1:1), 1% N2, 2% B27 supplement, 200 U/mL penicillin, 100 μg/mL streptomycin and 1% fetal bovine serum (FBS, #10270-160, Invitrogen, Carlsbad, CA, USA). At day 8, cells were fixed with 4% PFA for 15 min, immunocytochemistry staining was performed as above. Anti-GFAP and anti-β-tubulin III antibodies were used as the primary antibodies. Alexa Fluor 488 and 594 conjugated donkey anti-rabbit/donkey anti-mouse IgG were used as secondary antibodies. Cell nuclei were counterstained with DAPI and visualized under a fluorescent microscope (Olympus BX-57, Olympus, Tokyo, Japan) equipped with a DP70 digital camera and the DP Manager (DP Controller, Olympus, Tokyo, Japan) software (Version 3.3.1.222). For the negative control, the primary antibody was replaced by blocking buffer.

### 4.6. Western Blot Analysis

NSCs at a density of 1 × 10^6^ cells/mL were seeded in 6-well plates. After treatment, cells were lysed by using lysis buffer [1 × RIPA lysis buffer with 2% protease inhibitor cocktail (#04693159001, Roche, Basel, Switzerland); 1% phosphatase inhibitor (#524625, Millipore, Billerica, MA, USA)] and collected in Eppendorf (Ep) tubes, and then centrifuged at 13,000 rpm at 4 °C for 15 min. After centrifugation, the supernatants were collected and the concentration of all proteins was measured using a BCA Protein Assay Kit (ThermoFisher, Waltham, MA, USA). Equal amounts of protein extracts (10 μg) were electrophoresed in 10% SDS-polyacrylamide gels and transferred to 0.22 μm polyvinylidene fluoride membranes (PVDF; #IPVH00010, Millipore, Billerica, MA, USA). After blocking with 5% nonfat milk for 2 h, the PVDF membranes were incubated overnight at 4 °C. Blots were probed with antibodies (Cell Signal Technology, Beverly, MA, USA) recognizing the phospho-STAT3 (Tyr705) (#9145), STAT3 (#12640), phospho-AKT (Ser473) (#4058), AKT (#9272), phospho-ERK1/2(#9101) and ERK1/2 (#4695). All antibodies were diluted 1:1000 in 5% BSA, except for phospho-STAT3 (Tyr705), which was diluted 1:2000 in 5% BSA. After washing with TBS-T buffer (1 × TBS, 0.1% [*v*/*v*] Tween 20), the membranes were incubated with the secondary antibodies (HRP conjugated anti-rabbit or anti-mouse IgG) for 2 h at room temperature. The detection of the bands was done by the ECL Detect Kit (#WBKLS0500, Millipore, Billerica, MA, USA) using an ECL imager (Bio-Rad, Hercules, CA, USA). The expression bands of target proteins were analyzed by ImageJ software (ImageJ 1.4, NIH, Bethesda, MD, USA). The housekeeping protein β-actin (1:5000; Proteintech, Rosemont, IL, USA) was used as an internal control.

### 4.7. Statistical Analysis

Independent experiments were repeated at least three times in each trial. Data were presented as mean ± SD and analyzed with the SPSS 18.0 software. A value of *p* < 0.05 was considered to be statistically significant. One-way ANOVA and Least Significant Difference (LSD) multiple comparisons test were used for multiple comparison analysis. GraphPad Prism 8.0 (GraphPad Software Inc, San Diego, CA, USA) software was used for plotting graphs.

## Data Availability

The data presented in this study are available upon reasonable request to the corresponding author.
